# 

**Published:** 1846-10

**Authors:** 


### WESTERN JOURNAL

### OF

### MEDICINE AND SURGERY.

### EDITORS.

---

DANIEL DRAKE, M. D.,

PROFESSOR OF PATHOLOGY AND THE PRACTICE OF MEDICINE
IN THE MEDICAL INSTITUTE OF LOUISVILLE:

LUNSFORD P. YANDELL, M. D.,

PROFESSOR OF CHEMISTRY AND PHARMACY IN THE SAME:

THOMAS W. COLESCOTT, M. D.

### TO THE SUBSCRIBERS OF THE LOUISVILLE JOURNAL OF MEDICINE AND SURGERY.

On looking over the subscription books of this publication, we find that many of the subscribers are in arrears, and some of them very largely. As this is the harvest time of doctors in the West and South, there could not be a better occasion to ask them to remit. Come, gentlemen, a goodly harvest you have had of chills and fevers, congestions and ague-cakes, pay us who cater for your instruction. Come, it's just as easy as to thrust a lancet into a tumor, nothing more than to enclose $5, $10, $20, $30, or $35, as the case may be. Come, you have the reputation of mosquitoes for thrusting your bills into other people's faces—pay your own.

PRENTICE & WEISSINGER,
*Publishers and Proprietors.*

RECEIPTS OF MEDICAL JOURNAL FOR AUGUST AND SEPT.

| | |
|---|---|
| Drs. Davenport & Allen, Lawrenceburg, Tenn. - - - - - | $5 00 |
| Dr. J. Barry, Clay Village, Ky., - - - - - - - - - | 5 00 |
| Dr. E. Clifford, Grove's P. O., Ind., - - - - - - - | 5 00 |

---

The WESTERN JOURNAL OF MEDICINE and SURGERY is published monthly by the undersigned, at the corner of Main and Fifth streets, Louisville, at $5 per annum, payable in advance. Each number contains 96 pages, making two volumes in the year of more than 550 pages.

Contributions to its pages by the Physicians of the Valley of the Mississippi, addressed to the Editors, are respectfully solicited.

Letters on business, to be addressed, postage paid, to the publishers.

*January*, 1844. PRENTICE & WEISSINGER.

